# Salud auditiva y exposición a ruido ambiental en población de 18 a 64 años de Bogotá, Colombia, entre el 2014 y el 2018

**DOI:** 10.7705/biomedica.7271

**Published:** 2024-05-30

**Authors:** Jenny Andrea Sierra, Leyder Mónica Montaña, Karla Yohanna Rugeles, María Teresa Sandoval, Wilson Sandoval, Karem Johanna Delgado, Jhon Jairo Abella

**Affiliations:** 1 Grupo de Investigación en Salud Ambiental de Bogotá, Subred Integrada de Servicios de Salud Sur Occidente - E.S.E., Bogotá, D. C., Colombia Grupo de Investigación en Salud Ambiental de Bogotá Subred Integrada de Servicios de Salud Sur Occidente - E.S.E Bogotá D. C Colombia; 2 Grupo de Investigación en Salud Ambiental de Bogotá, Subred Integrada de Servicios de Salud Norte - E.S.E., Bogotá, D. C., Colombia Grupo de Investigación en Salud Ambiental de Bogotá Subred Integrada de Servicios de Salud Norte - E.S.E Bogotá D. C Colombia; 3 Grupo de Investigación en Salud Ambiental de Bogotá, Subred Integrada de Servicios de Salud Centro Oriente - E.S.E., Bogotá, D. C., Colombia Grupo de Investigación en Salud Ambiental de Bogotá Subred Integrada de Servicios de Salud Centro Oriente - E.S.E Bogotá D. C Colombia; 4 Grupo de Investigación en Salud Ambiental de Bogotá, Secretaría Distrital de Salud, Bogotá, D. C., Colombia Grupo de Investigación en Salud Ambiental de Bogotá Secretaría Distrital de Salud Bogotá D. C Colombia

**Keywords:** percepción auditiva, pérdida auditiva, ruido, efectos del ruido, pruebas auditivas, exposición a riesgos ambientales, Auditory perception, hearing loss, noise, noise effects, hearing tests, environmental exposure

## Abstract

**Introducción.:**

La salud auditiva es un tema de interés en salud pública que afecta la calidad de vida y que puede afectarse por la exposición continua al ruido, un factor de riesgo que genera síntomas auditivos y extraauditivos.

**Objetivo.:**

Identificar el estado de salud auditiva de adultos que viven en Bogotá, y su asociación con factores de exposición a ruido ambiental, individuales y otológicos.

**Materiales y métodos.:**

Se realizó un estudio transversal mediante el análisis de una base de datos con 10.311 registros, obtenidos entre los años 2014 y 2018, producto de una encuesta estructurada de percepción de ruido y tamizaje auditivo. Se hizo un análisis descriptivo bivariado y una regresión logística binaria.

**Resultados.:**

El 35,4 % de los participantes presentó disminución auditiva. En el componente de percepción: 13,0 % refirió no escuchar bien, 28,8 % informó síntomas extraauditivos, 53,3 % tenía antecedentes otológicos, y 69,0 % manifestó molestia por ruido extramural. En la regresión logística, las variables más asociadas con disminución auditiva fueron: de las ambientales, vivir en zonas de mayor ruido (OR = 1,50) (IC_95%_: 1,341,69); de las individuales, ser hombre (OR = 1,85) (IC_95%_: 1,64-2,09) y la edad (por cada año de vida, el riesgo de disminución auditiva aumentó 6 %); y de las otológicas, tener antecedente de síntomas otológicos (OR = 1,86) (IC_95%_: 1,66-2,08).

**Conclusiones.:**

La disminución auditiva es multicausal en la población evaluada. Los factores que aumentan su prevalencia son incremento de la edad, ser hombre, tabaquismo, medicamentos ototóxicos, vivir en zonas de mayor exposición a ruido y presentar síntomas extraauditivos.

## Abstract (en)

**Introduction.:**

Hearing health is a public health concern that affects the quality of life and can be disturbed by noise exposure, generating auditory and extra-auditory symptoms.

**Objective.:**

To identify the hearing health status in adults living in Bogotá and its association with environmental noise exposure and individual and otological factors.

**Materials and methods.:**

We conducted a cross-sectional study using a database with 10,311 records from 2014 to 2018, consigned in a structured survey of noise perception and hearing screening. We performed a descriptive, bivariate, and binary logistic regression analysis.

**Results.:**

Of the included participants, 35.4% presented hearing impairment. In the perception component, 13.0 % reported not hearing well; 28.8 % had extra-auditory symptoms, 53.3 % informed otological antecedents and 69.0 % presented discomfort due to extramural noise. In the logistic regression, the variables with the highest association for hearing impairment were living in noisy areas (OR = 1.50) (95% CI: 1.34-1.69), being male (OR = 1.85) (95% CI: 1.64-2.09), increasing age (for each year of life, the risk of hearing impairment increased 6%), and having history of extra-auditory symptoms (OR = 1.86) (95% CI: 1.66-2.08).

**Conclusions.:**

Hearing impairment is multi-causal in the studied population. The factors that promote its prevalence are increasing age, being male, smoking, ototoxic medications, living in areas with high noise exposure, and extra-auditory symptoms.

La salud auditiva y comunicativa se define como la capacidad efectiva y sana del ser humano para oír y entender, y está ligada a la función de comunicarse por medio del lenguaje. Esta capacidad depende de las estructuras y de la fisiología del oído -que es el órgano de la audición- y del cerebro; además, del grado de maduración del individuo y del ambiente sociocultural en el que se desenvuelve ^1^. En el oído se inicia el proceso para la comunicación con el medio ambiente, en la cual los sonidos no solo son percibidos, sino también, comprendidos y discriminados ^2^.

Según el Ministerio de Salud y Protección Social, una audición saludable consiste en "escuchar de manera natural los sonidos del ambiente, habla y lenguaje" ^3^, para que el ser humano desarrolle y potencie sus capacidades. Por lo tanto, una audición inadecuada afecta la calidad de vida y el desarrollo pleno de la persona ^1^. La disminución de la agudeza auditiva (hipoacusia) o la pérdida de la audición se presentan en humanos cuando el umbral de audición en ambos oídos es mayor o igual a 20 decibeles (dB) ^4^.

Las causas que pueden llevar a una pérdida auditiva son diversas: eventos adversos relacionados con el embarazo, la genética, el nacimiento, infecciones del oído, exposición a sonidos fuertes o consumo de medicamentos ototóxicos; estas dos últimas son las más comunes de pérdida de audición en los adultos. Estas causas pueden evitarse en la vida adulta, mediante estrategias de salud pública utilizadas a lo largo del curso de la vida. Estas consisten en limitar la exposición a ruidos, escuchar sin riesgos y vigilar la posible ototoxicidad de los medicamentos. Estas acciones, junto con una adecuada higiene otológica, pueden ayudar a mantener una buena audición y disminuir los riesgos de perderla ^4^^,^^5^.

Según lo reportado en el 2019 por la Organización Mundial de la Salud (OMS), más del 5 % de la población mundial (432 millones de adultos y 34 millones de niños) padece discapacidad por pérdida de la audición y 1.100 millones de jóvenes entre 12 y 35 años de edad están en riesgo de sufrirla por su exposición al ruido en contextos recreativos ^6^. En el plano nacional, en el año 2015, se estimó que cinco millones de personas presentaban problemas auditivos (11 % de la población del país) y que, entre la población de 25 a 50 años laboralmente activa, la prevalencia de pérdida de la audición por exposición a ruido era del 14 % ^7^. En Bogotá, según el registro para la localización y caracterización de personas con discapacidad, entre octubre del 2002 y octubre del 2018, la prevalencia de alteraciones permanentes en los oídos fue del 14,1 % (33.066 personas) ^8^.

Como se ha mencionado, uno de los factores de riesgo de la pérdida auditiva es el ruido, entendido como el exceso de sonido que altera las condiciones normales del ambiente en una zona determinada ^9^. El ruido ha representado un problema ambiental para el ser humano desde la antigüedad ^10^ y, actualmente, el creciente interés en este asunto ha permitido caracterizar algunas de las fuentes emisoras de ruido, como el sistema de transporte, las actividades económicas y antrópicas, y los ruidos generados por la naturaleza ^9^.

Colombia cuenta con normas para el control de ruido, emitidas desde el Ministerio de Salud, en las cuales se establecen los niveles máximos permitidos según la zona (residencial, comercial, industrial y de tranquilidad) y la hora del día. En horario diurno, los niveles oscilan entre 45 y 70 dB y, en horario nocturno, entre 45 y 75 dB ^11^.

Asimismo, el Ministerio de Ambiente, Vivienda y Desarrollo Territorial expidió la norma nacional 627 de 2006, sobre "emisión de ruido y ruido ambiental" ^12^, en la cual se definen los estándares máximos permitidos de emisión de ruido con la ponderación A (adaptada a la percepción del oído humano) para diferentes sectores. Las zonas residenciales o exclusivamente destinadas al uso habitacional tienen un estándar máximo de ruido diurno de 65 dB (A) y, en la noche, de 55 dB (A); las zonas con usos industriales tienen un estándar máximo permitido diurno de 75 dB (A) y uno nocturno de 70 dB (A).

Los efectos en la salud auditiva por exposición a ruido son múltiples; uno de ellos es el desarrollo de hipoacusia, que puede ser progresiva al cabo de los años, según el grado y la duración de la exposición ^13^. En la literatura científica, se ha documentado el comportamiento de la hipoacusia en adultos y su asociación con factores ocupacionales. Uno de los estudios indica que los grupos con gran riesgo de exposición al ruido ocupacional son los militares y los trabajadores de construcción y agricultura; otro, realizado en China, estableció que los trabajadores de las industrias de transporte, minería y manufactura típica, estuvieron ocupacionalmente expuestos a un ruido de 98,6 ± 7,2 dB (A). Estas investigaciones referencian entre el 5 y el 21 % trabajadores con pérdida auditiva ^14^^-^^16^.

Por otro lado, las pérdidas auditivas se han relacionado con factores propios de la vejez, con una prevalencia en aumento a partir de los 70 años, aproximadamente ^17^^,^^18^.

Los estudios de pérdida auditiva con abordajes desde la exposición al ruido ambiental son escasos, pero se encontraron algunos a nivel nacional. En uno de ellos, se buscó determinar la relación de los factores de riesgo asociados con la pérdida auditiva neurosensorial en población adulta y se encontró que la exposición al ruido estaba asociada significativamente (p = 0,017) [Serpa C, Arenas, W. Factores de riesgo asociados a pérdida auditiva neurosensorial en población adulta atendida en la IPS Fonomedical del municipio de Sincelejo en el primer semestre del año 2022 (trabajo de grado). Sincelejo: Universidad de Sucre; 2023).

En Bogotá, también se han realizado estudios similares, como el de Hernández *et al.* sobre la exposición a ruido ambiental de docentes de Fontibón y Engativá, en el cual encontraron pérdida auditiva en el 27,7 % de la población estudiada ^19^; y el de Quiroz *et al.,* sobre los efectos auditivos y neuropsicológicos por exposición a ruido ambiental en escolares, en el que la pérdida auditiva se presentó en el 14,8 % ^20^.

Teniendo en cuenta el contexto descrito y la escasa información sobre la afectación y percepción del ruido ambiental en la población bogotana, en este estudio se buscó caracterizar la salud auditiva en adultos y su posible relación con la contaminación por ruido ambiental y otros factores de exposición, con el fin de aportar en la construcción de estrategias de prevención e intervenciones que permitan orientar la toma de decisiones para mejorar la salud auditiva y comunicativa de esta población.

## Materiales y métodos

### Tipo de estudio y participantes

Se realizó un estudio de tipo transversal mediante el análisis de 10.311 registros recolectados entre el 2014 y el 2018 mediante una encuesta estructurada de percepción de ruido y tamizaje auditivo, aplicada dentro del proceso de vigilancia de salud ambiental de la línea de aire, ruido y radiación electromagnética de la Secretaría Distrital de Salud de Bogotá. Se incluyeron adultos entre los 18 y los 64 años, a quienes se les hizo tamizaje auditivo (audiometría) y encuesta de percepción.

Se realizó un muestreo no probabilístico, por conveniencia, en el que se abordó la población objetivo en las diferentes localidades del Distrito Capital, quienes aceptaron ser incluidos y la accesibilidad para continuar con el desarrollo de la intervención. Se identificaron zonas de mayor y menor exposición a ruido, de acuerdo con la caracterización local realizada por un profesional en ingeniería ambiental y mapas de ruido entregados por la Secretaría Distrital de Ambiente. La población caracterizada cumplió con los criterios de inclusión de edad (18 a 64 años) y tiempo de residencia (dos años o más en la misma unidad habitacional). Los criterios de exclusión utilizados fueron mujeres gestantes o lactantes y personas con alguna discapacidad que les impidiera contestar la encuesta.

### Recopilación de datos e instrumentos

Los datos recopilados corresponden a los obtenidos entre los años 2014 y 2018, mediante una encuesta de percepción de ruido estructurada con dos categorías de respuesta (sí o no) realizada en la unidad habitacional del participante. En esta se indagó por antecedentes personales y familiares, enfermedades, antecedentes otológicos, condiciones de salud, consumo de medicamentos, hábitos relacionados con la salud auditiva y la percepción frente a la exposición a la contaminación por ruido.

Por otro lado, un profesional en fonoaudiología procedió a practicar el examen físico para determinar la integridad funcional y anatómica del conducto auditivo externo, identificar posibles anomalías o alteraciones que pudieran condicionar el resultado de la prueba, y evaluar el estado del pabellón, el conducto y la membrana timpánica para descartar la presencia de tapones de cerumen, cuerpos extraños o estrecheces del canal auditivo. Cuando se presentaron casos de condiciones inflamatorias o infecciosas (otitis) o afección de las vías respiratorias altas, el participante no se incluyó en la intervención.

Para el registro de la audiometría por vía aérea, se tuvieron en cuenta las frecuencias establecidas en el audiograma: 250, 500, 1.000, 2.000, 3.000, 4.000, 6.000 y 8.000 Hz. El funcionamiento del audiómetro se verificó con una calibración biológica al inicio de la jornada (por lo menos, una vez en la semana).

### Procesamiento de los datos

Inicialmente, la base contenía 10.400 registros, que se redujeron después de la limpieza y el tratamiento de datos, la eliminación de duplicados o por incumplimiento de los criterios de inclusión y la ausencia de datos, entre otros. La base de datos definitiva contenía 10.311 observaciones.

Se digitalizó la información de todos los exámenes de tamizaje auditivo y las encuestas de percepción, y, posteriormente, se revisó la congruencia de las respuestas en medio físico y digital, para asegurar la calidad de los datos.

### Variables

Como variable dependiente, se seleccionó el resultado de la audiometría de tamizaje, que se calificó como: con disminución auditiva (mayor de 20 dB en dos o más de las frecuencias evaluadas entre 250 y 8.000 Hz) o sin disminución auditiva (menor de 20 dB). La variable independiente principal fue el grado de exposición a ruido en la zona de residencia, dividida en dos categorías: mayor exposición (residencia ubicada en zonas con nivel de ruido mayor o igual a 65 dB) y menor exposición (residencia ubicada en zonas con nivel de ruido menor a 65 dB). Para esta categorización, se utilizaron los mapas de ruido de la Secretaría Distrital de Ambiente, la caracterización previa de la zona realizada por un profesional en ingeniería ambiental y la evidencia reportada en la literatura científica.

Además de la encuesta de percepción, se incluyeron otras variables independientes relacionadas con la pérdida auditiva, como: la toma regular de medicamentos ototóxicos, el uso recreacional de audífonos, el uso de motocicleta, la asistencia a discotecas, el hábito de jugar al tejo, el de fumar, el antecedente de familiares con pérdida auditiva, el examen físico anormal del conducto auditivo externo y de la membrana timpánica en uno o en ambos oídos (la prueba de Mantel-Haenszel permitió identificar la confusión y analizar la variación en los coeficientes e intervalos de confianza de las membranas timpánicas y los conductos auditivos externos), la presencia de síntomas otológicos, la percepción de ruido en la vivienda, la molestia del ruido al desarrollar actividades intramurales o extramurales, y la afectación de la salud por exposición al ruido. También, se analizaron variables sociodemográficas como el sexo y la edad.

### Análisis estadístico

Se hizo un análisis descriptivo de las variables del estudio. Se calcularon las frecuencias absolutas y relativas para las variables nominales, y las medidas de tendencia central y dispersión para la variable cuantitativa de edad.

En el análisis bivariado, se calcularon las prevalencias y razones de prevalencia (RP) para la variable dependiente de disminución auditiva y las variables independientes. Se usó la prueba de c^2^, se computaron los intervalos de confianza del 95 % y se estableció la significancia estadística con valores de probabilidad inferiores a 0,05. Se consideraron como posibles variables explicativas, la zona residencial de mayor exposición al ruido (> 65 dB) y las demás mencionadas.

Finalmente, se llevó a cabo un análisis multivariado mediante una regresión logística binaria para estimar las razones de probabilidad *(odds ratio,* OR) ajustadas, con intervalos de confianza del 95 % y valores de probabilidad inferiores a 0,05. Para la selección de variables, se utilizó el método por pasos hacia adelante de Wald y la pertinencia de cada una en el modelo. Se incluyeron las variables que fueron estadísticamente significativas en el análisis bivariado (p < 0,05). La información se procesó con el *software* R, versión libre 2023.12.1+402.

### Consideraciones éticas

Los participantes firmaron un consentimiento informado en el cual se les explicaba la finalidad de la encuesta de percepción, la práctica de la otoscopia y la audiometría, el manejo de la información y su confidencialidad.

Los sujetos del estudio autorizaron el uso de sus datos por parte de la Secretaría Distrital de Salud para fines investigativos o académicos, con el compromiso de la protección de su identidad, privacidad e intimidad, de acuerdo con lo previsto en el decreto 1377 de 2013 que reglamenta la ley 1581 de 2012 (Política de protección de datos personales). El presente estudio contó con la aprobación del Comité de Ética de Investigación de la Secretaría Distrital de Salud, registrado en la tabla maestra de investigaciones con el código SDSCTI20230003.

## Resultados

Entre el 2014 y el 2018, se realizaron 10.311 encuestas de percepción y tamizajes auditivos a personas entre los 18 y los 64 años, con un promedio de edad de 44 años (desviación estándar, DE = ± 13,7 años). Las mujeres fueron 6.984 (67,7 %) y los hombres, 3.327 (32,3 %). Todos los participantes residían en Bogotá y llevaban viviendo en la unidad habitacional 14,7 años, en promedio (DE = ± 12,9 años).

La afectación de la salud se midió considerando la función auditiva y la presencia de síntomas auditivos y extraauditivos. Según el tamizaje, la frecuencia de disminución auditiva fue del 35,4 %, con mayor afectación en ambos oídos; sin embargo, solo el 13,0 % del total de encuestados percibió no escuchar bien. Para las frecuencias altas de 4.000, 6.000 y 8.000 Hz, el 20,5 % de los participantes presentaron disminución auditiva en el oído izquierdo y, el 19,3 %, en el oído derecho; el 28,8 % reportó síntomas extraauditivos. Cabe resaltar que el 53,3 % de las personas refirieron antecedentes de enfermedades o síntomas otológicos.

En relación con la exposición al ruido, el 69,0 % de las personas reportaron molestias por el ruido proveniente de fuentes extramurales; y frente a los hábitos que pueden afectar la audición, el de mayor frecuencia fue el uso de audífonos (26,8 %) (cuadro 1).

**Cuadro 1:** Variables descriptivas ambientales, individuales y otológicas de la población participante

Variables de exposición	Total de encuestados	
	(N = 10.311)	
Variables ambientales	n (%)	n (%)
	Mayor exposición	Menor exposición
	(≥ 65 dB)	(< 65 dB)
Zona de exposición a ruido en la vivienda	5.321 (51,6)	4.990 (48,4)
	Sí	No
¿Percibe ruido en la vivienda?	6.972 (67,6)	3.339 (32,4)
¿Le molesta el ruido proveniente de fuentes extramurales?^a^	7.113 (69,0)	3.198 (31,0)
Variables individuales	n (%)	n (%)
Sexo	Hombres	Mujeres
	3.327 (32,3)	6.984 (67,7)
	Sí	No
Uso recreacional de audífonos	2.760 (26,8)	7.551 (73,2)
Uso de moto	848 (8,2)	9.463 (91,8)
Concurrencia a discotecas	1.369 (13,3)	8.942 (86,7)
Concurrencia a tejo	562 (5,5)	9.749 (94,5)
¿Fuma o fumó?	1.546 (15,0)	8.765 (85,0)
¿Tiene algún familiar con pérdida auditiva?	1.246 (12,1)	9.065 (87,9)
¿Presenta síntomas extraauditivos por ruido?^b^	2.968 (28,8)	7.343 (71,2)
Variables otológicas	n (%)	n (%)
	Anormal	Normal
Examen físico del conducto auditivo externo del oído derecho^+^	1.179 (11,4)	9.132 (88,6)
Examen físico del conducto auditivo externo del oído izquierdo^+^	1.154 (11,2)	9.157 (88,8)
Examen físico de la membrana timpánica del oído derecho+	1.438 (13,9)	8.873 (86,1)
Examen físico de la membrana timpánica del oído izquierdo^+^	1.371 (13,3)	8.940 (86,7)
Antecedente de síntomas otológicos^c^	5.496 (53,3)	4.815 (46,7)
	Sí	No
¿Ha recibido tratamiento con medicamentos ototóxicos por más de dos meses?^d^	368 (3,6)	9.943 (96,4)
¿Siente que escucha bien?	8.967 (87,0)	1.344 (13,0)
¿Siente que escucha mejor por algún oído?	2.649 (25,7)	7.662 (74,3)
¿Por cual oído escucha mejor?		
Derecho	1.557 (58,8)	
Izquierdo	1.092 (41,2)	
Resultado del tamizaje auditivo (audiometria)	n (%)	n (%)
	Sí	No
Disminución auditiva según el resultado del tamizaje	3.654 (35,4)	6.657 (64,6)
Disminución auditiva de las frecuencias 4.000 a 8.000 en el oído izquierdo	2.118 (20,5)	8.193 (79,5)
Disminución auditiva de las frecuencias 4.000 a 8.000 en el oído derecho	1.994 (19,3)	8.317 (80,7)
	Unilateral	Bilateral
Lateralidad de la disminución auditiva	1.034 (28,3)	2.620 (71,7)
Disminución auditiva de las frecuencias 4.000 a 8.000	2.538 (61,7)	1.574 (38,3)

Fuente: Subdirección de Salud Pública, Secretaría Distrital de Salud. Línea aire, ruido y radiación electromagnética (2014-2018).

^+^ El resultado de la prueba de Mantel-Haenszel muestra que no hay confusión y, al hacer análisis de la variación de los coeficientes y los intervalos de confianza, no varían los resultados.

^a^ Actividades industriales, discotecas/bares, tráfico aéreo o terrestre, iglesias, pregoneo, comercio e instituciones educativas

^b^ Presencia de tres síntomas o más de los siguientes: irritabilidad, ansiedad, agotamiento físico, cefalea, dificultad de concentración e insomnio

^c^ Vértigo, otorrea, otitis, acúfenos, prurito y otalgia

^d^ Los medicamentos indagados fueron: furosemida, gentamicina, antituberculosos y aspirina.

En el análisis bivariado se usó la prueba de c^2^ con intervalos de confianza del 95 % y valores de probabilidad inferiores a 0,05. Se encontró que los factores ambientales más relacionados con un aumento en la prevalencia de la disminución auditiva fueron vivir en una zona de mayor exposición a ruido (> 65 dB) y percibir molestia por ruido proveniente de fuentes extramurales. En cuanto a los factores de riesgo otológico, los principales fueron el consumo de medicamentos ototóxicos por más de dos meses y la presencia de anormalidades en la membrana timpánica de ambos oídos. Respecto a las variables individuales, los hombres tuvieron mayor prevalencia de disminución auditiva en comparación con las mujeres, al igual que los participantes que fuman o fumaron en comparación con aquellos que no lo hicieron (cuadro 2).

Se aplicó el modelo de regresión logística con el método de selección de variables por pasos hacia adelante *(forward)* de Wald ^21^. En él, se incluyeron las variables que presentaron significancia estadística en el análisis bivariado (p < 0,05). Como resultado, se encontró que percibir ruido en la vivienda, hábito de tejo y examen físico anormal del conducto auditivo externo de ambos oídos no fueron estadísticamente significativas, por lo cual no se incorporaron en el modelo final (cuadro 2).

Se concluyó que el modelo de regresión logística binaria es apropiado, dada la validación de los supuestos de linealidad, independencia, multicolinealidad y bondad de ajuste. Las variables ambientales con mayor asociación para el resultado de disminución auditiva fueron vivir en una zona de exposición a ruido mayor de 65 dB (OR = 1,50) (IC_95%_: 1,34-1,69). En las variables individuales, ser hombre presentó la mayor asociación (OR = 1,85) (IC_95%_: 1,64-2,09) y la variable edad indicó que, por cada año de vida, el riesgo de presentar disminución auditiva aumentaba un 6 %. En cuanto a las variables otológicas, se observó que la mayor asociación con la disminución auditiva fue la del antecedente de síntomas otológicos como vértigo, otorrea, otitis, acúfenos, prurito y otalgia (OR = 1,86) (IC_95%_: 1,66-2,08) (cuadro 2).

**Cuadro 2:** Resultado del análisis bivariado y la regresión logística

Variables de exposición	Total de encuestados (N = 10.311)	Prevalencia de la disminución auditiva según las variables de exposición	Análisis bivariado	Regresión logística
Variables ambientales	n (%)	n (%)	RP (IC_95%_)	OR ( IC_95%_)
Tipo de exposición a ruido				
Mayor exposición	5.321 (51,6)	2.114 (39,7)	1,28 (1,22-1,35)	
(> 65 dB)			p = 0,000	1,50 (1,34-1,69)
Menor exposición	4.990 (48,4)	1.540 (30,9)		p = 0,000
(< 65 dB)				
¿Le molesta el ruido proveniente de fuentes extramurales?^a^				
Sí	7.113 (69,0)	2.738 (38,5)	1,34 (1,26-1,43)	1,20 (1,01-1,41)
No	3.198 (31,0)	916 (28,6)	p = 0,000	p = 0,030
¿Percibe ruido en la vivienda?				
Sí	6.972 (67,6)	2.633 (37,8)	1,23 (1,16-1,31)	-
No	3.339 (32,4)	1.021 (30,6)	p = 0,000	
Variables individuales	n (%)	n (%)	RP (IC_95%_)	OR (IC_95%_)
Sexo				
Hombres	3.327 (32,3)	1.393 (41,9)	1,29 (1,22-1,36)	1,85 (1,64-2,09)
Mujeres	6.984 (67,7)	2.261 (32,4)	p = 0,000	p = 0,000
¿Presenta síntomas extraauditivos por ruido?^b^				
Sí	2.968 (28,8)	1.271 (42,8)	1,32 (1,25-1,39)	1,32 (1,18-1,48)
No	7.343 (71,2)	2.383 (32,5)	p = 0,000	p = 0,000
¿Fuma o fumó?				
Sí	1.546 (15,0)	618 (40,0)	1,15 (1,07-1,23)	1,19 (1,02-1,39)
No	8.765 (85,0)	3.036 (34,6)	p = 0,000	p = 0,022
Edad^c^			-	1,06 (1,06-1,07)
Concurrencia a tejo				p = 0,000
Sí	562 (5,5)	266 (47,3)	1,36 (1,24-1,49)	-
No	9.749 (94,5)	3.388 (34,8)	p = 0,000	
Variables otológicas	n (%)	n (%)	RP **(IC**_95%_ **)**	OR **(IC**_95%_ **)**
¿Ha presentado algún síntoma otológico?^d^				
Sí	5.496 (53,3)	2.258 (41,1)	1,41 (1,34-1,49)	1,86 (1,66-2,08)
No	4.815 (46,7)	1.396 (29,0)	p = 0,000	p = 0,000
Examen físico de membrana timpánica del oído derecho				
Anormal^e^	1.438 (13,9)	724 (50,3)	1,52 (1,43-1,61)	1,60 (1,33-1,92)
Normal	8.873 (86,1)	2.930 (33,0)	p = 0,000	p = 0,000
Examen físico de membrana timpánica del oído izquierdo				
Anormal	1.371 (13,3)	694 (50,6)	1,52 (1,44-1,62)	1,42 (1,18-1,72)
Normal	8.940 (86,7)	2.960 (33,1)	p = 0,000	p = 0,000
¿Ha recibido tratamiento con medicamento ototóxico por más de dos meses?^f^				
Sí	368 (3,6)	210 (57,1)	1,64 (1,50-1,80)	1,34 (1,03-1,74)
No	9.943 (96,4)	3.444 (34,6)	p = 0,000	p = 0,027
Examen físico del conducto auditivo externo del oído derecho				
Anormal^g^	1.179 (11,4)	512 (43,4)	1,26 (1,17-1,35)	-
Normal	9.132 (88,6)	3.142 (34,4)	p = 0,000	
Examen físico del conducto auditivo externo del oído izquierdo				
Anormal	1.154 (11,2)	511 (44,3)	1,29 (1,20-1,38)	-
Normal	9.157 (88,8)	3.143 (34,3)	p = 0,000	

RP: razones de prevalencia; IC: intervalo de confianza; OR: ***odds ratio***

Fuente: Subdirección de Salud Pública, Secretaría Distrital de Salud. Línea aire, ruido y radiación electromagnética (2014-2018).

^a^ Actividades industriales, discotecas/bares, tráfico aéreo o terrestre, iglesias, pregoneo, comercio e instituciones educativas

^b^ Presencia de tres síntomas o más de los siguientes: irritabilidad, ansiedad, agotamiento físico, cefalea, dificultad de concentración e insomnio

^c^ Variable numérica analizada en la regresión logística

^d^ Vértigo, otorrea, otitis, acúfenos, prurito y otalgia

^e^ Las anormalidades contempladas en membrana timpánica fueron: opaca, perforada, abultada, retraída o presencia de placas blancas. ^f^ Los medicamentos indagados fueron: furosemida, gentamicina, antituberculosos y aspirina.

^g^ Las anormalidades contempladas para el conducto auditivo externo fueron: tapón de cerumen total, tapón de cerumen parcial y cuerpo extraño.

## Discusión

Este estudio tuvo como objetivo identificar el estado de salud auditiva de adultos entre los 18 y los 64 años residentes en Bogotá, y su asociación con la exposición a ruido ambiental y factores individuales y otológicos. El tamizaje auditivo (audiometría) es usado en programas de detección temprana como una acción prioritaria, que debe realizarse en el transcurso de la vida, para la detección precoz, el diagnóstico oportuno y la atención integral de la hipoacusia, la discapacidad auditiva o ambas ^22^. De los 10.311 participantes, se obtuvo una frecuencia de disminución auditiva del 35,4 % aunque solo el 13.0 % percibía no escuchar bien. No obstante, la percepción del sonido es muy subjetiva ^23^. Algunas personas con pérdida auditiva niegan y tratan de ocultar esta disminución, ya que comúnmente se asocia con el envejecimiento ^24^, y otras puede que simplemente no la perciban ^25^.

En la regresión logística, la influencia de la variable sexo en la disminución auditiva es importante, ya que los hombres presentaron 1,85 veces más probabilidades de tenerla en comparación con las mujeres. Al respecto, en algunos trabajos, como el desarrollado por Cruickshanks *et al.* ^26^, en el que examinaron los factores de riesgo y los de protección contra enfermedades otológicas en una cohorte de 16.415 hispanos o latinos de cuatro ciudades de Estados Unidos, se encontró que los hombres son un 66 % más propensos a padecer una pérdida auditiva que las mujeres. Asimismo, en un estudio reciente desarrollado en China por Wang *et al.* ^27^, en el que participaron 1.140 hombres y 1.140 mujeres con edades entre los 18 y los 60 años, se informó que la prevalencia de pérdida auditiva de alta frecuencia es mayor en hombres (34,4 %) que en mujeres (13,8 %). Sin embargo, como señalan otros reportes, no está claro si esto se debe a que los hombres tienen más probabilidades que las mujeres de estar expuestos a ruidos peligrosos en el lugar de trabajo.

En este estudio dirigido a población de 18 a 64 años, se encontró que, por cada año de vida de los participantes, aumentó un 6,0 % la disminución auditiva. Hernández *et al.* ^2^ informaron que las pérdidas auditivas pueden aumentar a partir de los 25 años debido a cambios fisiológicos propios de la edad; no obstante, esto puede variar a causa de factores endógenos o exógenos de cada individuo. En el estudio de carga global de morbilidad asociada con la prevalencia de pérdida auditiva y años vividos con discapacidad (llevado a cabo entre 1990 y 2019) ^28^, se encontró que el 62.1 % de las personas con pérdida auditiva eran mayores de 50 años.

Por otro lado, en una revisión de la literatura realizada por Cavallieri *et al.* ^29^, se resaltó el efecto ototóxico del cigarrillo y se evidenció que algunos estudios reportaban peores umbrales de audición en los fumadores en comparación con los no fumadores. A su vez, Dawes *et al.* ^30^ informaron que los fumadores actuales tienen mayores probabilidades de pérdida de audición que los que nunca habían fumado (OR = 1,15) (IC_95%_: 1,09-1,21). En el presente estudio, fumar o haber fumado también estuvo asociado significativamente con disminución auditiva (OR = 1,19) (IC_95%_: 1,02-1,39).

De las variables ambientales estudiadas, se observó que el residir en zonas de mayor exposición al ruido y la molestia por el ruido proveniente de actividades extramurales son factores de riesgo para la disminución auditiva. En relación con esto, Hong *et al.* ^31^ reportaron que los cambios en la audición están asociados con varios factores de riesgo modificables, incluida la exposición al ruido. Este factor ha cobrado importancia en las ciudades, según lo reportado por Hernández *et al.* ^*2*^, ya que el desarrollo de la industria trae consigo un incremento considerable del ruido, como el emitido por las maquinarias, los medios de transporte, etc., que contaminan el ambiente y son un elemento de riesgo para la salud de la población.

Paiva *et al.* encontraron que el 48,4 % de los 225 encuestados en São Paulo (Brasil) refirieron molestia relacionada con el ruido ^32^. Esto es similar a lo reportado en Querétaro (México), por Peñaloza *et al.* ^33^, donde el 40,48 % de los encuestados no se encontraba satisfecho con el ruido percibido en su colonia o barrio. Lo anterior también fue manifestado por los participantes de este estudio: el 67,6 % refirió percibir ruido en la vivienda y, de estos, el 57,5 % informó molestia en el desarrollo de sus actividades dentro de ella.

En el presente estudio, las variables otológicas estuvieron asociadas con disminución auditiva, pues las personas que presentaron algún síntoma, como acúfenos, vértigo, otalgia, otitis, otorrea o prurito, tuvieron mayor prevalencia de disminución auditiva. Al respecto, Swartz ^34^ indicó que los acúfenos y el vértigo se pueden asociar con hipoacusia, mientras que Morales *et al.* ^35^, reportaron que el 43 % de los pacientes que consultaron con antecedente de *tinnitus,* una enfermedad compleja y multifactorial, presentaron hipoacusia en la audiometría.

En el contexto nacional, en el trabajo ya mencionado de Serpa y Arenas, se informó que los participantes del estudio consultaron en su mayoría por pérdida auditiva (36,8 %; n = 85), *tinnitus* (24,7 %; n = 57) y vértigo (20,3 %; n = 47), y que uno de los factores de riesgo asociados con la pérdida auditiva neurosensorial es el vértigo (p = 0,031) (Serpa C, Arenas W. Factores de riesgo asociados a pérdida auditiva neurosensorial en población adulta atendida en la IPS Fonomedical del municipio de Sincelejo en el primer semestre del año 2022 (trabajo de grado). Sincelejo: Universidad de Sucre; 2023).

En el estudio transversal de Pérez *et al.* ^36^, se buscó identificar los factores asociados con la otitis media crónica en 344 pacientes de dos centros de referencia en otología de Bogotá, a los que se les practicó evaluación otoscópica y audiométrica. Los pacientes presentaban algunos síntomas recurrentes de otitis media crónica, como pérdida de audición, secreción, *tinnitus* y anomalías en el equilibrio, relacionados con un deterioro significativo de la calidad de vida debido a dificultades de comunicación social y un menor rendimiento laboral.

En la misma línea, en el estudio realizado por Peñaranda *et al.* ^37^, cuyo objetivo fue describir la gravedad del *tinnitus* en pacientes con otitis media crónica, se encontraron alteraciones en los resultados audiométricos y de la calidad de vida; por esto, los autores recomiendan desarrollar estudios que utilicen métodos estandarizados para la evaluación de los factores asociados con la calidad de vida y la carga financiera de estas enfermedades.

Por otra parte, las anormalidades encontradas en el examen de membrana timpánica de ambos oídos también están asociadas con disminución auditiva. Según revisiones, como la de Muñoz *et al.* ^38^, los pacientes con hipoacusia conductiva generalmente tienen alteraciones en la membrana timpánica, pero también, pueden encontrarse tapones de cera, dermatitis y micosis.

El tratamiento con medicamentos ototóxicos se asoció con la disminución auditiva de los participantes, específicamente, con el uso de furosemida, gentamicina, antituberculosos y aspirina; este último fue el de mayor consumo por parte de los participantes (2,9 %; n = 302). La información disponible sobre esta asociación es diversa: Swartz indica que ciertos medicamentos pueden provocar súbitamente una hipoacusia bilateral, es decir, una pérdida rápida e inexplicable de la audición. Medicamentos ototóxicos como los aminoglucósidos -estreptomicina y gentamicina- pueden destruir las células pilosas del órgano de Corti y provocar una sordera permanente; los salicilatos y diuréticos -como la furosemida o el ácido etacrínico- pueden provocar una pérdida transitoria de la audición a dosis altas; y otros, como el quimioterápico cisplatino, también están asociados con ototoxicidad ^34^^,^^39^. Como se ha descrito en la literatura, la ototoxicidad depende de varios factores, como la concentración del fármaco en el oído interno, las características del paciente, el uso concomitante de otros fármacos ototóxicos y el tratamiento previo con aminoglucósidos ^40^.

Otra de las variables analizadas en el presente estudio, fueron los síntomas extraauditivos que también se asociaron con disminución auditiva. El 42,8 % de las personas con alteraciones en el examen de tamizaje, refirieron tres o más síntomas de los siguientes: irritabilidad, ansiedad, agotamiento físico, cefalea, dificultad de concentración e insomnio. Los hallazgos del presente estudio se relacionan con lo descrito por Quispe *et al.* en Perú, ^41^, quienes reportaron que el 26 % de los 380 participantes de la ciudad de Juliaca había presentado dolores de cabeza y, el 24 %, estrés, entre otros.

Por su parte, el estudio de Rahman *et al.* ^42^, realizado en Pakistán, reveló que los participantes de las ciudades de Jhang y Chiniot presentaron problemas de salud relacionados con el ruido, como molestia (53; 51 %), depresión (45; 47 %), mareos (61; 65 %), dolor de cabeza (67; 64 %), hipertensión (71; 56 %), pérdida de audición (53; 56 %), estrés fisiológico (65; 65 %), insomnio (81; 84 %) y *tinnitus* (70; 62 %).

Como se ha descrito, el aumento de la prevalencia de la disminución auditiva en la población estudiada en Bogotá está asociada con diferentes factores, como la edad, el sexo masculino, el hábito de tabaquismo, el uso de medicamentos ototóxicos y vivir en zonas de mayor exposición a ruido. Entre los afectados, es más frecuente encontrar síntomas otológicos y extraauditivos, y alteraciones en la membrana timpánica.

Los resultados obtenidos en el presente estudio contribuyen a la investigación de las causas de la disminución auditiva y pueden aportar a la generación de campañas enfocadas en la promoción del autocuidado de la salud auditiva, principalmente, en aquellas personas con factores de riesgo ambientales, individuales u otológicos, como los mencionados.

Se resalta la importancia de implementar tamizajes auditivos periódicos en la población de mayor riesgo, educar en la prevención de la automedicación de fármacos ototóxicos en la comunidad y diseñar estrategias para que estos solo sean prescritos por personal médico. Se destaca la importancia de desarrollar estudios con audiometrías clínicas para obtener resultados más sensibles, entre ellos, los relacionados con el promedio tonal auditivo.

A nivel intersectorial y sectorial, es pertinente promover la reducción del ruido, mediante estrategias para el control de las fuentes de emisión y la sensibilización sobre el ruido ambiental (capacitaciones, eventos masivos sobre la problemática del ruido, normas, prácticas que generen menor contaminación por ruido, difusión en medios de comunicación), dirigidas a los sectores comercial, industrial y educativo, espacios públicos y actores de movilidad, entre otros.

Una de las limitaciones de esta investigación es que, al tratarse de un estudio transversal, no es posible establecer una secuencia temporal entre la exposición y el resultado que permita determinar causalidad. Asimismo, puede existir el riesgo de sesgo de recuerdo o memoria al mencionar los antecedentes de salud; sin embargo, tener una encuesta estructurada con categorías de respuesta cerrada, ayudó al control de dicho sesgo.

Este estudio tuvo un muestreo no probabilístico por conveniencia, en zonas de mayor y menor exposición a ruido de acuerdo con los mapas de Secretaría Distrital de Ambiente, en el cual la población aceptó participar voluntariamente.

Por otra parte, el uso de la información de una base con datos del 2014 al 2018 considera variables con condiciones que, en la actualidad, pueden haber cambiado por dinámicas ambientales, demográficas, epidemiológicas (como la pandemia de COVID-19) y comportamentales. Por lo anterior, se requiere desarrollar nuevos estudios que permitan documentar la situación actual, local y nacional.

No obstante, este trabajo presenta un panorama de la salud auditiva asociada con el ruido ambiental en la población adulta de Bogotá y constituye un insumo innovador para profundizar en las problemáticas relacionadas con este contaminante y priorizar intervenciones en las poblaciones más afectadas.
